# An Unusual Presentation of Primary Lymphoma of the Ilium

**DOI:** 10.1155/2014/509837

**Published:** 2014-09-08

**Authors:** Mohamad Gouse, Viswanath Jayasankar, Manika Alexander

**Affiliations:** ^1^Department of Orthopaedics, Unit 1, Christian Medical College, Vellore, India; ^2^Department of Pathology, Christian Medical College, Vellore, India

## Abstract

Primary bone lymphoma involving the pelvic bone is a rare entity. It does not have distinct clinical features or radiological features that are diagnostic. Biopsy is the gold standard investigation. We present a case of primary pelvic lymphoma with initial histopathological features of chronic osteomyelitis. Upon further clinical follow-up, repeat biopsy of the lesion revealed features of B-cell Non-Hodgkin's lymphoma, thus emphasizing the need for a high index of suspicion and close clinical follow-up. This case is presented for the diagnostic dilemma and the unique feature of lymphomatous lesion mimicking chronic osteomyelitis in its early stages.

## 1. Introduction

Primary bone lymphoma represents approximately 3% of all malignant bone tumors and 5% of all Non-Hodgkin lymphomas [1]. Most osseous lymphomas present with similar characteristics such as an increased incidence in men, usually in fifth and sixth decades, with common sites being long bones (50%) and most being of B-cell lineage [2]. The incidence of lymphoma in innominate bones is very rare [3]. Radiologically, lymphomas do not have characteristic features and can mimic other tumorous conditions. We report a case of primary lymphoma of the ilium, masquerading as chronic osteomyelitis. We present this case because of its rarity of the location and diagnostic dilemma.

## 2. Case Report

**Figure 1:**
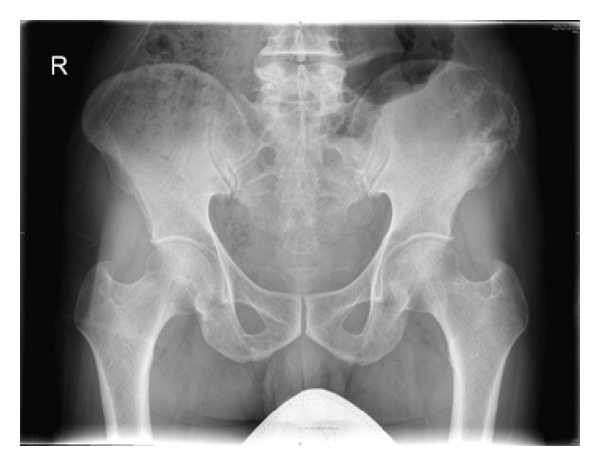
Plain radiograph of Pelvis showing an osteolytic lesion with indistinct margins, cortical erosion, and narrow transitional zone over the left ilium.

**Figure 2:**
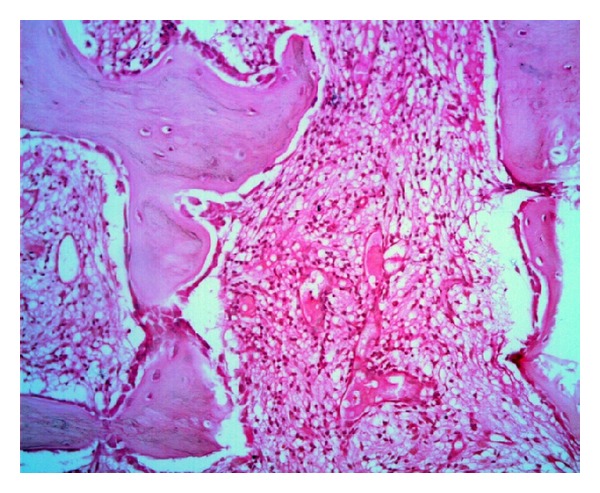
Photomicrograph of chronic osteomyelitis ilium showing trabeculae of cortical, cancellous, and scanty necrotic bone with intervening inflammatory granulation tissue densely infiltrated by lymphocytes and histiocytes (H&E stain, ×20).

A 65-year-old gentleman presented to the out-patient department with three-month history of continuous, dull aching pain in the left iliac region, radiating to the left thigh. He did not have constitutional symptoms like fever, weight loss, and loss of appetite. Initial examination revealed a 3 × 4 cm tender swelling with indistinct margins localized over the lateral aspect of the left iliac crest. There was increase in temperature and erythema. General examination was unremarkable except for pallor. There was no localized or generalized lymphadenopathy. Blood investigations revealed normal haemogram, ESR of 20 mm/hour and C-reactive protein of 34 mg/dl. Plain radiographs revealed an osteolytic lesion with indistinct margins, cortical breach, moth eaten appearance, and narrow transitional zone (Figure 1). Bone scan showed isolated uptake in the left ilium. In view of age and location, differential diagnosis included primary bone tumor and indolent infection. A needle biopsy was performed. The histopathological examination showed trabeculae of cortical, cancellous, and scanty necrotic bone with intervening inflammatory granulation tissue densely infiltrated by lymphocytes and histiocytes consistent with nonspecific chronic osteomyelitis (Figure 2). No tumor markers or special strains were done. In spite of the biopsy suggesting chronic osteomyelitis, the cultures were negative for mycobacterium, fungal elements, and bacterial elements. Further, there was no conclusive clinicoradiological correlation. Hence, it was suggested that the patient underwent an open biopsy proceed excision of the iliac lesion. However, the patient decided to defer any surgical procedure. Hence, he was started on a course of empirical antibiotics and advised to review regularly.

**Figure 3:**
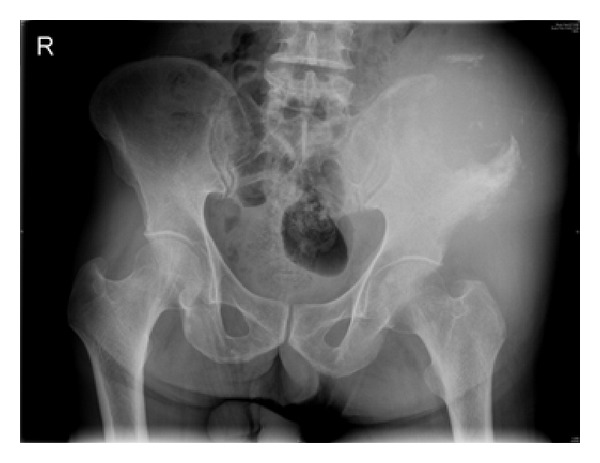
Plain radiograph of pelvis showing a permeative lesion in the left ilium with significant cortical destruction.

**Figure 4:**
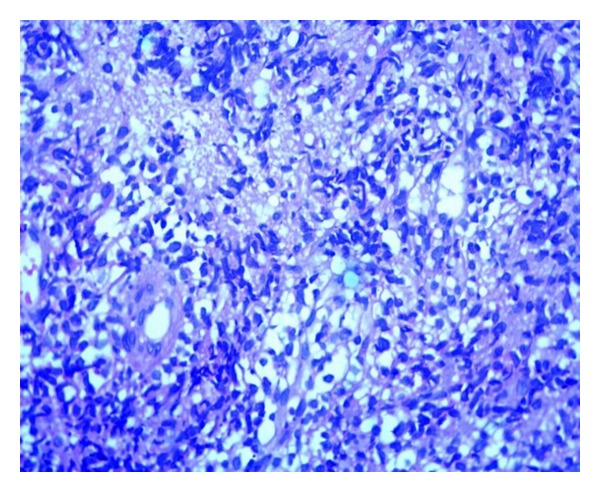
Photomicrograph of diffuse large B-cell lymphoma of ilium showing sheets of medium-sized atypical lymphoid cells (H&E stain, ×20).

**Figure 5:**
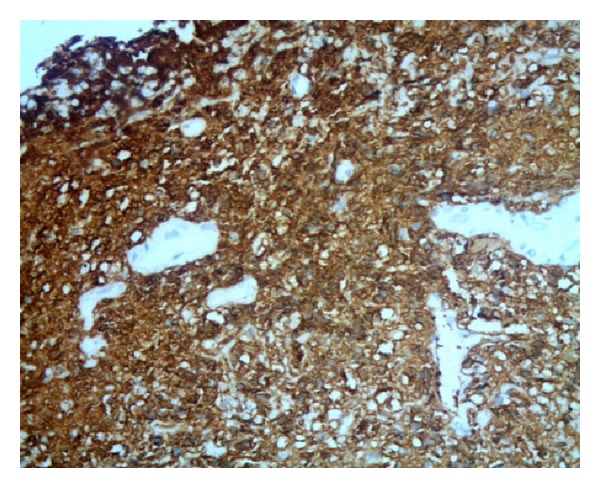
Photomicrograph of diffuse large B-cell lymphoma of ilium showing CD20 positive atypical lymphoid cells (CD20 stain, ×40).

At his follow-up visit, the patient appeared pale and had significant loss of weight. The swelling had increased to size of 10 × 10 cm, with extent up to the left inguinal region. The left lower limb was edematous. There was no localized lymphadenopathy. Hip movements were painfully restricted. Plain radiographs of pelvis showed a permeative lesion with significant periosteal reaction over the left iliac crest (Figure 3). The chest radiograph, computed tomography of chest, and ultrasound abdomen were negative for any lesion. An open biopsy was performed revealing cores of scanty partially crushed tumor composed of sheets of medium-sized round cells with round to oval nuclei, small nucleoli, scant to moderate amounts of cytoplasm, and areas of necrosis. Immunohistochemistry revealed tumor cells that were positive for CD20 and Bcl-6 and negative for Tdt, Bcl-2, MUM-1, CD5, CD23, and CD3. A diagnosis of high grade B cell Non-Hodgkin's lymphoma was made (Figures 4 and 5). Medical oncology opinion was obtained and the tumor was staged as Ann Arbor Ia–Extra nodal, with risk stratification being low-intermediate. The patient was then planned for a combined treatment modality with chemotherapy and radiotherapy.

## 3. Discussion

First established as a clinical entity in 1939, primary lymphoma of the bone is defined as “lymphoma presenting in an osseous site with no evidence of disease elsewhere for at least six months after diagnosis” [4]. It presents with constant pain, as a palpable mass with soft tissue extension or with pathologic fracture. Systemic “B” symptoms may also be present at the time of diagnosis [5]. Iliac lesions are difficult to diagnose in the initial stages; further, Limb et al. showed there is mean delay of eight months in the diagnosis of lymphoma of the bone [3, 6]. Skeletal involvement is rare and limited to the long bones and axial skeleton, with femur being the most common site. Though intrapelvic, nonosseous lymphoma has been reported; involvement of the innominate bones has been described sparingly in world literature [7].

The paucity of systemic “B” symptoms and relative rarity of lymphoma in the ilium made us consider another differential diagnosis. Clinically, lymphoma of the bone can mimic the presentation of other small round cell tumors such as myeloma and rarely present with innocuous symptoms analogous to that of osteomyelitis [2, 8].

The diagnosis of lymphoma presenting as a primary bone lesion can cause a dilemma as there are no pathognomonic radiological findings. In plain radiographs, lymphomas have variable and nonspecific appearance ranging from being near normal to a diffusely permeative lesion with soft tissue extension. The lytic-destructive pattern is the most common (70%) and is usually associated with an aggressive periosteal reaction. Cortical breakthrough, an ominous sign, is present in less than 25% of early lymphomas. About one-third of primary lymphomas present with a blastic-sclerotic pattern (13.6%) or with subtle near normal findings (5.8%) [7]. CT and MRI scans though helpful in assessment and staging of the tumor are neither conclusive nor diagnostic. MRI is the preferred modality for imaging of suspicious lesions with T2-weighted images revealing areas of low signal intensity [9]. A pattern of extensive marrow disease and surrounding soft tissue masses but without extensive cortical destruction is a reported characteristic of an osseous lymphoma [10, 11].

The radiological picture in our patient at first presentation demonstrated a lytic, radiolucent, pelvic lesion with cortical erosion which represents the possibility of infection. However, radiographs at follow-up demonstrated a permeative lesion with ill-defined margins and soft tissue extension, strongly suggestive of a malignancy. The radiological appearance of a primary bone lymphoma continues to evolve, emphasizing the need for a regular and meticulous follow-up [2].

Closed or open biopsy and histopathological examination are the gold standard diagnostic investigation [12]. Needle biopsy of Non-Hodgkin's lymphoma may not be adequate as it may provide tissues with crush artifact and areas of decalcification. Needle biopsy of lesions with a significant soft tissue component or areas of inflammatory changes has higher false negative rates [13]. The diagnostic yield of a needle biopsy is more dependent on the size of the specimen obtained than on the gauge of the needle. Wu et al. in their study showed that minimum of four specimens for soft tissue lesions and three specimens for bony lesions were most favourable for a diagnostic yield [14]. The initial biopsy performed in our patient showed the features of osteomyelitis. There was no cellular atypia or any features to suspect otherwise. Hence, further characterization was not done. At his follow-up visit, unrelenting symptoms prompted a need to reconsider the diagnosis. Non-Hodgkin's lymphoma appears most commonly with large cells with irregular cleaved nuclei and prominent nucleoli surrounded by reticulin fibers. Histological sections reveal a monotonous infiltrate of mixed small and large lymphocytes with associated crush artifact and area of extensive necrosis. Immunohistochemical stains reveal positive staining for LCA (CD45), CD20, CD10, Bcl-2, Bcl-6, and CD15 and positivity for Ki-67(MIB-1) [15]. A repeat biopsy confirmed the presence of the abovementioned features of a diffuse large B-cell lymphoma in both microscopy and immunohistochemistry.

The lag time for primary lymphoma of bone to manifest its clinical features poses a diagnostic difficulty, thus emphasizing the need for a high degree of suspicion when clinical and histopathological findings do not tally. Delay in diagnosis can have serious consequences and the need to obtain an urgent diagnosis has been stressed [16].

To our knowledge, this is the first report of an innominate bone lymphoma presenting as chronic osteomyelitis. This has been reported due to the unusual location of this tumor, the ambiguity in clinical symptoms, and the diagnostic dilemma thus caused.

## Key Topics

Non-Hodgkin's lymphoma presenting with features of chronic osteomyelitis is a rare, poorly described entity and can pose a diagnostic dilemma.
